# Neuropsychic effects of organic solvents: a review of the Tunisian literature

**DOI:** 10.1192/j.eurpsy.2023.2020

**Published:** 2023-07-19

**Authors:** H. Ziedi, S. Chemingui, D. Brahim, J. Rjeb, N. Mechergui, N. Ladhari, I. Youssef

**Affiliations:** occupational health department, Charles Nicolle Hospital, Tunis, Tunisia

## Abstract

**Introduction:**

Organic solvents (OS) constitute a considerable occupational risk in industrial environment. Long-term exposure can cause several neuropsychic manifestations which are the subject of several studies conducted in Tunisia.

**Objectives:**

To identify the main psychological disorders in workers exposed to OS in Tunisia and to determine the occupational sectors at risk.

**Methods:**

This is a review of the Tunisian literature, focusing on studies carried out on OS in the workplace and published in the form of articles or defended in the form of theses or dissertations in medical faculties over a ten-year period.

**Results:**

The total number of employees was 9 499 of which 4 259 were exposed to OS. The total number of companies studied was 169 with 164 occupational pathology cases. The most studied sectors were the chemical industry (20 studies) and the leather industry (21 studies). Atmospheric concentration of OS was measured in 20 studies. A mixture concentration index was calculated in 70 cases, it did not comply with the standard in 30 cases. Pathologically, the syndrome of acquired intolerance to organic solvents had a prevalence of 1.8% to 38.2%, while the syndrome of psychic dependence had a prevalence of 8.9% to 35.3%. The prevalence of organic psycho-syndrome ranged from 0.8% to 26.5%.

**Conclusions:**

Despite the methodological differences of the studies, this work can contribute to the evaluation of the extent of the problem posed by OS in the workplace for a possible implementation of an adapted preventive approach.

**Disclosure of Interest:**

None Declared

